# Impact of COVID-19 pandemic on diagnostic pathology in the Netherlands

**DOI:** 10.1186/s12913-022-07546-w

**Published:** 2022-02-09

**Authors:** M. L. F. van Velthuysen, S. van Eeden, S. le Cessie, M. de Boer, H. van Boven, B. M. Koomen, F. Roozekrans, J. Bart, W. Timens, Q. J. M. Voorham

**Affiliations:** 1 Department of Pathology, Erasmus Medical Center, Rotterdam, Netherlands; 2 Department of Clinical Epidemiology, Leiden University Medical Center, Leiden, Netherlands; 3 Department of Pathology, University Medical Center Utrecht, Utrecht, Netherlands; 4 Department of Pathology, Netherlands Cancer Institute, Amsterdam, Netherlands; 5 Laboratory of Pathology Oost Nederland (LABPON), Hengelo, Netherlands; 6 Dutch Society of Pathology (NVVP), Leiden, Netherlands; 7 Department of Pathology and Medical Biology, University Medical Center Groningen, Groningen, Netherlands; 8 Pathologisch Anatomisch Landelijk Geautomatiseerd Archief (PALGA), Houten, Netherlands

**Keywords:** COVID-19, Workload, Pathology diagnostics, Malignancy rate, Cytopathology

## Abstract

**Background:**

The COVID-19 pandemic has a huge impact on healthcare provided. The nationwide pathology registry of the Netherlands, PALGA, offers an outstanding opportunity to measure this impact for diseases in which pathology examinations are involved.

**Methods:**

Pathology specimen numbers in 2020 were compared with specimen numbers in 2019 for 5 periods of 4 weeks, representing two lockdowns and the periods in between, taking into account localization, procedure and benign versus malignant diagnosis.

**Results:**

The largest decrease was seen during the first lockdown (spring 2020), when numbers of pathology reports declined up to 88% and almost all specimen types were affected. Afterwards each specimen type showed its own dynamics with a decrease during the second lockdown for some, while for others numbers remained relatively low during the whole year. Generally, for most tissue types resections, cytology and malignant diagnoses showed less decrease than biopsies and benign diagnoses. A significant but small catch-up (up to 17%) was seen for benign cervical cytology, benign resections of the lower gastro-intestinal tract, malignant skin resections and gallbladder resections.

**Conclusion:**

The COVID-19 pandemic has had a significant effect on pathology diagnostics in 2020. This effect was most pronounced during the first lockdown, diverse for different anatomical sites and for cytology compared with histology. The data presented here can help to assess the consequences on (public) health and provide a starting point in the discussion on how to make the best choices in times of scarce healthcare resources, considering the impact of both benign and malignant disease on quality of life.

**Supplementary Information:**

The online version contains supplementary material available at 10.1186/s12913-022-07546-w.

## Background

During the first spike of the COVID-19 pandemic extensive numbers of COVID-19 patients put an enormous strain on healthcare services worldwide. In ensuring enough hospital capacity for severely ill COVID-19 patients less urgent medical treatment was often put on hold. Moreover, patients avoided or delayed seeking care for other health issues [1–4].

While medical staff working in the frontline were stretched to their limits, the workload for some other healthcare workers diminished. Among them were pathologists who received less specimens than usual, especially during lockdown [5–11]

At first glance this might just seem an obvious consequence of the situation, but investigating the reduced diagnostic volume in detail may provide important insights. The number and kind of specimens received in pathology laboratories reflect an important part of patientcare given, so taking stock of the diagnostic workload for pathologists may help understand what happened clinically. Identifying the most affected areas in pathology may reveal healthcare choices that were either implicitly or explicitly made during the crisis and may be a good starting point for evaluating their long-term effects. This is why some have propagated a monitoring of pathology specimens [12].

The Netherlands is a country particularly suitable to provide data on how the diagnostic volume in pathology laboratories changed during the COVID-19 pandemic, because it has a nationwide database named PALGA (Pathologisch Anatomisch Landelijk Geautomatiseerd Archief), which registers all pathology reports since 1991 [13].

By making use of the PALGA database this study assesses the nationwide changes in specimen numbers for the different areas in pathology throughout 2020, in contrast to previous reports that were restricted to single institutions or specific modalities/tissue types [7, 9, 10, 14]. The present study not only covers the first spike of the COVID-19 pandemic in spring 2020, but also the periods after the first lockdown and the second spike in autumn. It is evaluated whether and how numbers decreased, whether and how quickly decreased numbers returned to normal and whether any catching up was seen.

## Methods

### Aim

Study the impact of the COVID-19 pandemic on diagnostic pathology in general to identify the most affected areas in pathology and the healthcare choices that were made. Thus creating a starting point for evaluating their long-term effects.

### Design

To examine the impact of governmental measures against the coronavirus on diagnostic volume, specimen numbers per week were calculated in five periods of four weeks (Table 1) e.g. weeks 13–16, 23–26, 33–36, 43–46 and 47–50.

**Table 1:** Periods used to measure effect of pandemic

Period 1	W13- W16	2020: 23/3 – 19/4 1^st^ lockdown and Easter 2019: 25/3 – 21/4
Period 2	W23- W26	2020: 1/6 – 28/6 after 1^st^ lockdown and Pentecost 2019: 3/6 – 30/6 Pentecost
Period 3	W33-W36	2020: 10/8 – 6/9 2019: 12/8 – 8/9
Period 4	W43-W46	2020: 19/10 – 15/11 start 2^nd^ lockdown 2019: 21/10 – 17/11
Period 5	W47-W50	2020: 16/11 – 13/12 2^nd^ lockdown 2019: 18/11 – 15/12

### Setting

The first Dutch COVID-19 patient was officially diagnosed on February 27^th^ 2020. In early March the first corona-related death was reported and in the following weeks the number of COVID-19 patients increased rapidly [15].

The government announced progressively strict measures to control the virus outbreak, resulting in an ‘intelligent lockdown’ from March 16^th^ to June 1^st^. People were allowed to leave their houses, but the lockdown rules were aimed at minimising social interaction. For example, schools, universities, gyms, restaurants, pubs, theatres and museums were closed, events were cancelled, people were urged to work from home, to avoid contact with others and not to use public transport unless absolutely necessary.

Hospitals had to postpone much of the non-urgent medical care to cope with the increasing numbers of COVID-19 patients. Doctors restricted the number of actual patient visits by implementing telemedicine (phone calls, video consulting) as much as possible. The Department of Health decided to pause the Dutch population screening programmes for colorectal carcinoma, breast carcinoma and cervical carcinoma from March 16^th^ to relieve the pressure on healthcare services. Screening for these malignancies was gradually resumed in the second half of May, the second half of June and July respectively.

On June 1^st^ the lockdown measures were lifted until the second spike of the COVID-19 pandemic announced itself with a rapid increase in the number of COVID-19 cases from September onwards. On October 14^th^ the government reintroduced measures similar to those of the first lockdown, although schools remained open. This changed on December 16^th^ when more restrictions were imposed: schools and non-essential shops had to close, resulting in a strict lockdown.

While healthcare services intended to continue non-COVID patientcare as much as possible during this period, the increasing number of COVID-19 patients and the drop-out of healthcare workers forced hospitals to downscale regular patientcare, although not as much as in spring.

### Materials and processes

In the Netherlands a nationwide database PALGA records all pathology reports since 1991, which are coded by localisation (organ, body part or tissue type), procedure and diagnosis [13].

Weekly case numbers of histological and cytological specimens from 2015–2020 were retrieved from the PALGA database, grouped by anatomical site, together with data on procedure (biopsy, resection) and dignity (benign versus malignant diagnosis) of the diagnosis.

The analyses on biopsies and resections contained lower specimen numbers than the total number of cases, because cases seen for revision or in consultation (and thus reported at least twice by different pathology laboratories) were not included.

For analyses on benign versus malignant not only cases seen for revision or in consultation were excluded, but also cases difficult to classify as benign or malignant, such as dysplasia, inadequate material and uncertain diagnosis. In cervical cytology PCR tests for HPV are reported separately from reports on morphologic examination and were therefore excluded.

### Statistical analysis

The observed numbers in 2020 were either compared with expected numbers (see more detailed explanation below) or with numbers of the same period of the previous year.

The expected numbers per week for 2020 were obtained from the average specimen numbers per week in the period 2015–2019, multiplied by a correction factor for changing trend over time. This correction factor was obtained by dividing the actual specimen numbers for week 2–10 of 2020 (pre-COVID-19) by the average numbers for week 2–10 of the 2015–2019 period.

To establish whether numbers of pathology specimens in 2020 were different from those in previous years the following strategies were followed:

1. The total numbers observed in 2020 (M) were compared to the expected numbers according to localisation, by calculating the ratio of observed versus expected numbers with corresponding 95% confidence interval, and expressing the ratio as percentage. When the 95% confidence interval did not contain the value 1 (100%), observed numbers were considered to deviate significantly from expected numbers. Asymptotic confidence intervals were used (mean ± 1.96 standard error), assuming that observed numbers (M) followed a Poisson distribution.
2. To compare numbers in subgroups by procedure (biopsy, resection) and by dignity of diagnosis (benign, malignant) the ratio of the numbers measured in 2020 (M_20_) and the numbers measured in 2019 (M_19_) was calculated. The confidence interval of this ratio was calculated, using that the standard error of a ratio of two independent Poisson distributed count variables is approximately equal to $\sqrt{\frac{{M}_{20}}{{{M}_{19}}^{2}}+ \frac{{{M}_{20}}^{2}}{{{M}_{19}}^{3}}} .$ The numbers measured in 2020 were considered to deviate significantly if the 95% confidence interval of the calculated ratios did not contain the value 1 (100%).

Both for strategy 1 and 2 ratios of less than 85% or more than 115%, if not significantly different, are highlighted as possibly clinically significant [16]. Analyses were not performed if there were less than 20 cases per week.

## Results

### General Pathology trends

Based on the data from previous years (2015–2019) the expected diagnostic workload for all Dutch pathology departments in 2020 was a total number of 2,975,469 cases. However, pathologists received only 82% of the expected number (2,427,960 cases). The sharpest drop was observed in period 1 during the first lockdown in spring: the number of reported specimens (78,465) was only 33% of the expected number (236,789).

For histological specimens expected versus measured numbers for 2020 were 1,895,580 versus 1,633,804 (86%); for cytology expected versus measured numbers were 196,619 versus 178,180 (90,63%). For cervical cytology specimens expected versus measured numbers were 326,841 versus 302,269 (92%).

### Trends in Cytology

Table 2 shows that in 2020 the relative decrease in numbers was most severe during the first lockdown, period 1 (week 13- week 16) in particular for specimens from cervix and breast (22% and 35% of expected respectively), for which national screening programmes were put on hold. In period 1 all areas of cytology showed a lockdown dip, with the most modest dip for pancreas cytology, where the number of examined specimens remained within the expected range (Table 2).

**Table 2:**
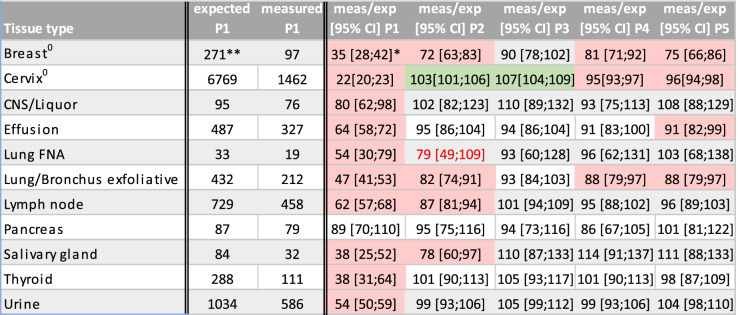
Cytology examinations per week according to tissue type during the 5 periods examined

^0^Tissues with national screening programme. ^**^Number of cases per week. ^*^Ratio expressed as % of observed versus expected numbers with, in brackets, confidence interval (mean ± 1.96 standard error), assuming that observed numbers follow a Poisson distribution. P1-P5 refers to periods mentioned in Table 1

Red and green boxes show situations where the confidence interval of the ratio of measured and expected does not contain 100% and is considered statistically significantly different. The red number shows a ratio of less than 85%, which might be clinically significant

**Table 3:**
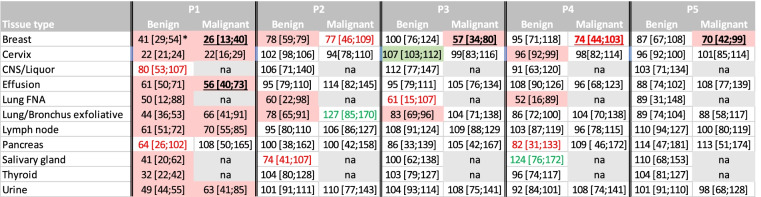
Benign and Malignant Cytology examinations in 2020 versus 2019 during the 5 periods examined

Ratio expressed as % of the numbers observed in 2020 and the numbers observed in 2019 with, in brackets, the confidence interval of this ratio, calculated using the standard error of a ratio of two independent Poisson distributed count variables. P1-P5 refers to periods mentioned in Table 1. Red and green boxes show situations where the confidence interval of the ratio of observed numbers in 2020 versus 2019 do not contain 100% and are considered statistically significantly different. Bold numbers show the situations where the relative decrease in malignant specimens is larger than for benign specimens. The red and green numbers show a ratio of less than 85% or more than 115%. For numbers of cytology examinations per week, all specimens, benign and malignant diagnoses, see supplementary table 1. na = not available (less than 20 specimens per week)

**Table 4:**
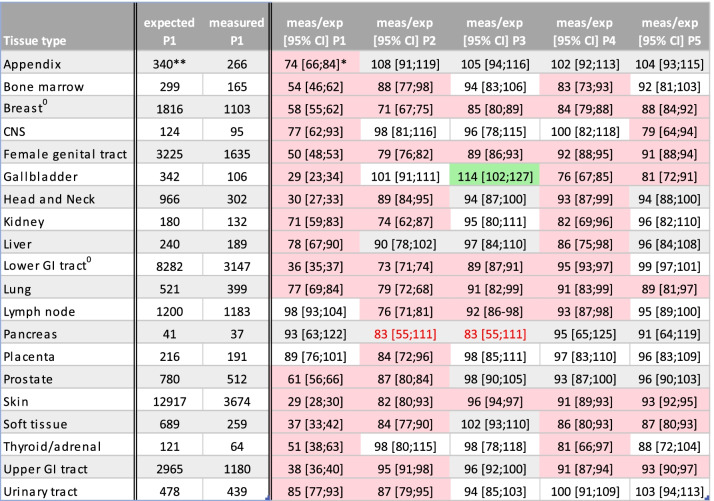
Expected versus measured numbers of histology examinations per week

^0^Tissues with national screening programme. ^**^Number of cases per week. ^*^Ratio expressed as % of the numbers observed in 2020 and the numbers observed in 2019 with, in brackets, the confidence interval of this ratio, calculated using the standard error of a ratio of two independent Poisson distributed count variables. P1-P5 refers to periods mentioned in Table 1. Red and green boxes show situations where the confidence interval of the ratio of measured and expected does not contain 100% and is considered statistically significantly different. The red numbers show a ratio of less than 85%, which might be clinically significant

**Table 5:**
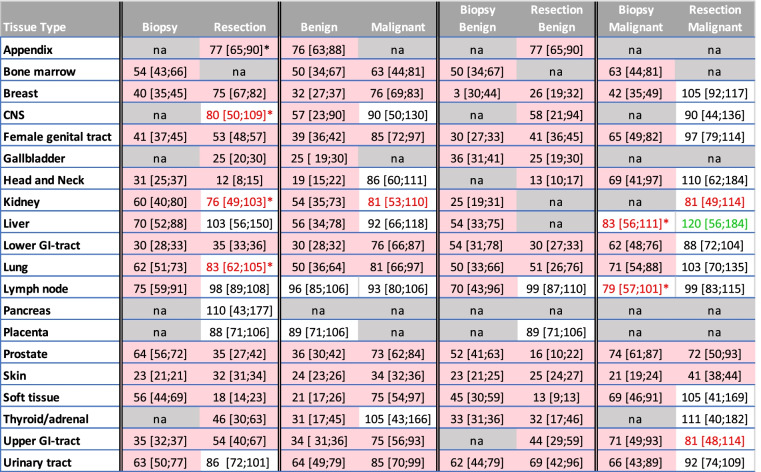
Histology examinations in 2020 versus 2019 week 13 – week 16

*Ratio expressed as % of the numbers observed in 2020 and the numbers observed in 2019 with, in brackets, the confidence interval of this ratio, calculated using the standard error of a ratio of two independent Poisson distributed count variables. Red boxes show situations where the confidence interval of the ratio of measured and expected does not contain 100% and is considered statistically significantly different. Red and green numbers show a ratio of less than 85% or more than 115%, which might be clinically significant. For numbers of histology examinations per week, biopsies, resections, benign and malignant diagnoses, see supplementary table 2. Analogous tables for periods 2–5 are given in supplementary tables 3–6. na = not available (less than 20 specimens per week)

In most areas (central nervous system (CNS), lung fine needle aspiration (FNA), salivary gland, thyroid and urine; Table 2) numbers recovered in period 2 and were within expected limits for the rest of the year. Numbers of cervix cytology showed a significant increase in periods 2 and 3 (after the first lockdown)with only a small dip during the second lockdown (periods 4 and 5). Numbers of lymph node and salivary gland cytology did not recover until period 3. For breast cytology, exfoliative cytology of bronchus/lung and for effusion specimens a second dip was seen in periods 4–5 (Table 2).

For most areas the decline in benign diagnoses was stronger than the decline in malignant diagnoses during the lockdown dip of period 1 (Table 3). However, for breast cytology and effusion specimens the number of malignant diagnoses decreased relatively stronger than the number of benign diagnoses. For breast cytology there was a decline in malignant diagnoses in all periods (74% in period 1; 23–43% in period 2–5).

The significant increase in cervical specimen numbers in period 2 and 3 could be attributed to benign cases (Table 3). For numbers of malignant exfoliative lung cytology in period 2 and for benign salivary gland cytology in period 4 the increase of more than 15% (Table 3) might suggest a catch-up.

### General trends for histologic specimens

For histology, the largest decrease was observed during the first lockdown for all tissues, except for lymph node, pancreas and placenta (Table 4).

The sharpest lockdown dip was seen for specimens from the skin, gallbladder (Table 4), head and neck, soft tissue and upper and lower gastrointestinal tract (29%-35% of expected).

The maximal decrease was much less in the next periods with a maximal decrease up to 71% of expected. Total numbers remained below expected throughout the year for breast, female genital tract, lung and skin. Numbers from the lower gastrointestinal tract remained below expected until period 5.

Numbers of appendiceal specimens recovered in period 2 and remained at expected levels during the rest of the year.

In other areas an initial recovery was seen in period 2 (CNS, gallbladder, liver, thyroid/adrenal gland) or period 3 (bone marrow, breast, head and neck, kidney) with a slighter drop during the second lockdown, mainly in period 4 (14–24% drop in period 4 vs 22–71% drop in period 1), but for some tissues also in period 5 (breast, gallbladder) or in period 5 only (CNS). For urinary tract and prostate, a recovery did not occur before period 3, but numbers remained within expected limits in period 4 and 5.

Although lymph node did not show a lockdown dip in period 1, numbers dropped significantly below expected in period 2, 3 and 4.

The only statistically significant catch-up in histology numbers was seen for gallbladder in period 3 (114%).

### Trends in biopsy versus resection and benign versus malignant

In the first lockdown period, the number of benign diagnoses dropped relatively more than the number of malignant diagnoses for most tissues (Table 5), except for lymph node samples for which no significant drop was seen for benign diagnoses in period 1 nor for malignant diagnoses in any period.

A similar pattern was seen for biopsies versus resections, where biopsies dropped more severely in period 1 than resections, except for head and neck, prostate and soft tissue for which the drop in resections was larger. Resections with a malignant diagnosis were spared in period 1 (Table 5).

**Fig. 1:**
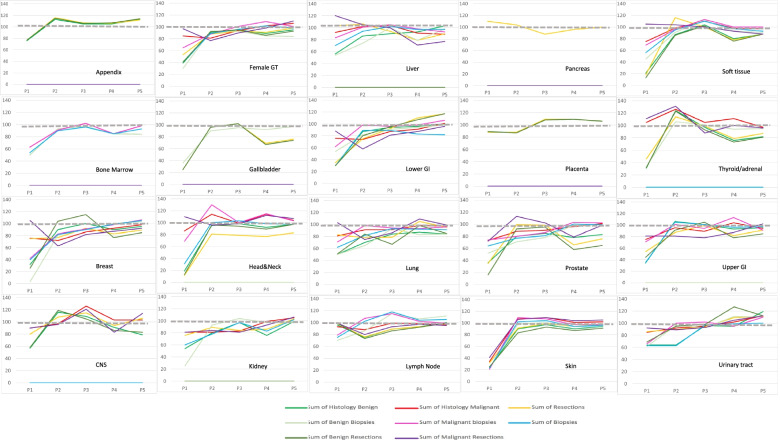
Ratio of observed average numbers per week of histology examinations in 2020 versus 2019 for biopsies, resections, benign and malignant specimens according to tissue type in all periods examined (period1-period5). Legend to Fig. 1: grey dotted line shows 100% level. Green lines show benign samples, bright green are all benign samples, light green biopsies, dark green resections. Purple and red lines show malignant samples, bright red are all benign samples, light purple biopsies, dark purple resections. Yellow lines are all resections. Blue lines are all biopsies

A statistically significant decrease of numbers in other periods than the first lockdown period was seen in at least one other period for benign diagnoses of most tissues (Fig. 1 and supplementary tables 3–7). A statistically significant reduction of malignant diagnoses in other periods than the first lockdown period was only seen for breast, lower GI-tract, female genital tract and prostate, mostly in period 2, immediately after the first lockdown. Only numbers of malignant breast diagnoses were affected in more periods (Fig. 1 and supplementary tables 3–7).

For many tissues the decrease during the second lockdown (periods 4 and 5) could be attributed to a decrease in specimens with a benign diagnosis (Fig. 1 and supplementary tables 5 and 6).

A statistically significant increase of specimen numbers (catch-up) was only seen for malignant skin specimens, especially resections, in period 3, between lockdowns (supplementary table 4 and Fig. 1) and for benign lower-GI tract resections in period 5 (supplementary table 6 and Fig. 1).

## Discussion

In 2020 the world faced a new reality when it had to cope with the COVID-19 pandemic. Many aspects of people’s lives were affected by the illness itself and the measures taken to restrain viral spread. Healthcare services struggled to find a balance between treating the large numbers of COVID-19 patients and continuing regular non-COVID patientcare.

The number of examined pathology specimens is an indirect measure for a part of the healthcare provided. The changes in specimen numbers may help understand which areas of clinical care were affected most during the pandemic and are at risk to suffer long term effects. The Netherlands has a nationwide database containing all pathology reports since 1991 (PALGA), which creates an outstanding opportunity to study this impact and to explore which areas were affected most, balancing single institutional reports [7–11, 14].

In line with the number of hospital admissions and number of persons dying from COVID-19, the strongest decline in specimen numbers was observed during the first spike of the pandemic. The decrease in this period was very high (67%), but total numbers remained below expected during the whole year (overall decrease of 18%). A second dip during the second lockdown was seen for some tissue types and procedures, although much less prominent. Moreover, not all specimen types were affected equally.

During the first lockdown national screening programmes for colorectal carcinoma, breast carcinoma and cervical carcinoma were paused. This explains the huge decline in cytology specimens from cervix and breast (78% and 65% respectively) and the strong decrease in histological specimens from the lower gastrointestinal tract (65%) in period 1 as was also reported in other regions [9, 17]. As screening was resumed after the first lockdown, cervical cytology numbers showed a catch-up in period 2 and 3. Colorectal biopsies containing a malignancy returned to expected levels after period 1, but breast biopsies from malignant lesions were below expected in period 2 as well. Moreover, numbers of resected malignancies remained lower than expected for both lower gastrointestinal tract and breast in period 2 and 3 and period 2–4 respectively.

Part of these numbers were previously reported by the Dutch Cancer Registry (Integraal Kankercentrum Nederland, IKNL), which reported a drop in the numbers of stage I colorectal carcinoma, early stages of breast cancer and breast carcinoma in situ due to the temporarily suspended screening programmes. Numbers of newly diagnosed patients returned to expected levels in autumn [18–20]

The decline in resected malignancies from lower gastrointestinal tract, female genital tract and breast after period 1 might not only reflect a lower number of cancer diagnoses. It might be that a choice for other (neoadjuvant) treatment modalities like radiotherapy and/or chemotherapy was made more frequently under the given circumstances, thus postponing surgery and putting less strain on intensive care facilities [2, 21–23]. However, a catch-up in numbers is not seen.

In the normal situation histology of the skin makes up the largest part of the diagnostic volume in Dutch pathology laboratories, but during the first lockdown numbers plummeted with 72%. Although biopsies and resections from benign skin disease remained low, a slight catch-up (between 5–9%) was seen for malignant skin disease after period 1. Skin care might belong to the clinical areas which are delayed relatively easily in times of crisis because many skin diseases are not or may not seem immediately life-threatening. Moreover, patients might delay seeking care for lesions that do not cause severe symptoms [24, 25].

In contrast to the areas discussed above, other areas seem hardly affected by the pandemic. It is not surprising that placentas belong to this category, but remarkably numbers for pancreas, central nervous system (CNS) and liver remained relatively stable as well. Considering that diseases of these organs are often treated in specialised tertiary care centres, it raises the question whether their relatively stable numbers result from a conscious choice to prioritise the treatment of certain diseases, whether the stable numbers are caused by the severity of presenting symptoms or whether the way in which COVID-19 patients were spread over the Dutch hospitals, dictated indirectly which non-COVID patientcare could be continued.

Apart from the areas discussed above, a general observation is that the number of specimens containing a malignancy decreased relatively less than the number of specimens with benign disease, suggesting that a serious effort was made to continue cancer care as much as possible. This is supported by the fact that the number of resections for malignancies dropped less than the number of biopsies. The decline in biopsy numbers might, although partly attributed to the pause in screening programmes, also be due to people’s reluctancy to seek medical care. The observation that cytology numbers were affected less than biopsy numbers might be explained by the slightly different role of cytology in the diagnostic process.

Catch-up in numbers after the first lockdown dip was minimal. It was only seen for histology and the maximum was 17% (for benign resections of the colon). In all other instances in which some catch up was observed (e.g. resections of malignant skin lesions) the catch-up was less than 10%.

Because the pandemic put a strain on intensive care availability, this indirectly affected surgical capacity. A Dutch research group developed a model to predict the health impact of postponing surgical procedures for both benign and malignant disease by estimating the disability-adjusted life-years per month of delay [26]. This model shows that 20 of the 23 surgical procedures for which a delay would have the strongest negative impact, were oncological. Our analyses show that surgery for malignant disease was relatively spared during the corona crisis, which seems a logical choice based on the data from the Gravesteijn study.

The situation for benign disease is more difficult to assess. Most of the surgical procedures for benign disease included in the study by Gravesteijn et al. do not produce pathology specimens. So similar studies/models for different procedures are needed to assess the impact of the decrease in benign specimens observed in our study, as in the study of Te Groen et al. [27]. The procedures involved in the catch-up seen for benign resections of the lower GI-tract in our study might be the follow-up of their results.

Although the PALGA-database covers pathology reports nationwide and many areas of patientcare, it obviously does not include all healthcare areas. Because the results of this study reflect the Dutch situation, it might in several respects be different from the situation in other countries and populations. Moreover, for some categories specimen numbers were too small for meaningful analysis. Despite these limitations the results of this study highlight remarkable changes in patientcare during the COVID-19 crisis in the western world with a population that has easy access to a sophisticated healthcare system. Evidently this will be different for instance in middle and low income countries with a different demography. The long term impact is yet unknown, but it raises important questions:

What is the consequence of the pause in national screening programmes? Will the experiences of the past year, with a decline (and thus at least partly delay) in the number of surgical interventions, lead to a shift in the use of different treatment modalities for certain diseases (e.g. chemoradiotherapy instead of surgery)? Will there be a worse outcome for patient with postponed cancer diagnosis and/or treatment? What is the impact on health and quality of life of delaying surgery (skin, soft tissue, breast, gallbladder, prostate) or biopsy diagnosis (colon) for benign disease? Should a new or continuing pandemic lead to different healthcare choices?

## Conclusions

The COVID-19 pandemic has a significant effect on pathology diagnostics, which in this paper is shown from a nationwide point of view for the whole year of 2020. This effect was most pronounced during the first lockdown but lasted in some areas until the end of 2020 and may reflect more durable changes. Partly the changes seem to reflect short time choices, such as pausing screening programs and less invasive diagnostic procedures e.g. cytology instead of biopsies. Moreover, in some fields only marginal reductions were seen, possibly reflecting the impossibility of reducing care in these areas, such as brain, bone marrow, pancreas and appendiceal pathology. This last one as opposed to that in other countries where the decrease of appendectomies was almost 50% [4]. Together with studies on the health impact of postponing surgical procedures [26], the data presented here can help to assess the consequences on (public) health and provide a starting point in the discussion on how to make the best choices in times of scarce healthcare resources, recognizing the impact of both benign and malignant disease on quality of life.

## Supplementary Information

**Additional file 1.**

## Data Availability

The data that support the findings of this study are available from PALGA but restrictions apply to the availability of these data, which were used under license for the current study, and so are not publicly available. Data are however available from the authors upon reasonable request and with permission of PALGA.

## Abbreviations

PALGA: Pathologisch Anatomisch Landelijk Geautomatiseerd Archief
PCR: Polymerase Chain Reaction
HPV: Human Papilloma Virus
COVID: Corona Virus Disease
CI: Confidence interval
FNA: Fine Needle Aspiration
CNS: Central Nervous System
GI: Gastro-Intestinal
IKNL: Intergraal Kankercentrun Nederland
RIVM: Rijks Instituut voor Volksgezondheid en Milieu
