# Supplementary material for: Impact of COVID-19 pandemic on diagnostic pathology in the Netherlands
Source: BMC Health Serv Res. 2022 Feb 9;22:166. doi: 10.1186/s12913-022-07546-w (PMC8826665; doi:10.1186/s12913-022-07546-w)
Supplement: Supplementary file 1 — Additional file 1. [file 12913_2022_7546_MOESM1_ESM.docx]

**Supplementary tables**

**Supplementary table 1. Number of cytology examinations per week (expected in 2020 or observed in 2019). All specimens, benign and malignant diagnoses**

| **Tissue type** | **All** | **Benign** | **Malignant** |
| --- | --- | --- | --- |
| Cervix | 6394 | 4994 | 278 |
| Urine | 945 | 843 | 78 |
| Lymph node | 671 | 317 | 206 |
| Effusion | 447 | 302 | 100 |
| Lung/Bronchus (exfoliative) | 401 | 306 | 74 |
| Thyroid | 265 | 154 | 8 |
| Breast | 251 | 141 | 62 |
| CNS/Liquor | 87 | 78 | 4 |
| Pancreas | 81 | 22 | 22 |
| Salivary gland | 78 | 49 | 4 |
| Lung | 31 | 21 | 11 |

Legend to supplementary table 1: For all specimen numbers the expected numbers for 2020 are given. For benign and malignant numbers the observed numbers of 2019. Cervix cytology accounts for 66% of total numbers, 70% of benign numbers, but for only 33% of malignant diagnoses.

**Supplementary Table 2. Mean number of histology examinations per week (expected in 2020 and observed in 2019). Total numbers, biopsies, resections, benign and malignant diagnoses**

Legend to supplementary table 2: For total numbers the expected numbers for 2020 are given. For biopsies, resections, benign, malignant numbers and combinations, the observed numbers of 2019. Dark-gray boxes are those with less than 20 cases per week and therefore not included in further analyses.

**Supplementary table 3. Ratio of observed average numbers per week in 2020 versus 2019 for biopsies, resections, benign and malignant specimens according to tissue type in week 23 – week 26, after first lockdown.**

Legend to supplementary table 3: ^*^Ratio of the numbers observed in 2020 and the numbers observed in 2019 with, in brackets, the CI of this ratio, calculated using the standard error of a ratio of two independent Poisson distributed count variables. Red boxes show situations where the confidence interval of the ratio of measured and expected does not contain 100% and is considered statistically significantly different. Red and green numbers show a ratio of less than 85% or more than 115%, which might be clinically significant. na = not available (less than 20 specimens per week).

**Supplementary table 4. Ratio of observed average numbers per week in 2020 versus 2019 for biopsies, resections, benign and malignant specimens according to tissue type in week 33 – week 36, between both lockdowns.**

Legend to supplementary table 4: ^*^Ratio of the numbers observed in 2020 and the numbers observed in 2019 with, in brackets, the CI of this ratio, calculated using the standard error of a ratio of two independent Poisson distributed count variables. Red and green boxes show situations where the confidence interval of the ratio of measured and expected does not contain 100% and is considered statistically significantly different. Red and green numbers show a ratio of less than 85% or more than 115%, which might be clinically significant. na = not available (less than 20 specimens per week).

**Supplementary table 5. Ratio of observed average numbers per week in 2020 versus 2019 for biopsies, resections, benign and malignant specimens according to tissue type in week 43 – week 46, during second lockdown.**

Legend to supplementary table 5: ^*^Ratio of the numbers observed in 2020 and the numbers observed in 2019 with, in brackets, the CI of this ratio, calculated using the standard error of a ratio of two independent Poisson distributed count variables. Red boxes show situations where the confidence interval of the ratio of measured and expected does not contain 100% and is considered statistically significantly different. Red and green numbers show a ratio of less than 85% or more than 115%, which might be clinically significant. na = not available (less than 20 specimens per week).

**Supplementary table 6. Ratio of observed average numbers per week in 2020 versus 2019 for biopsies, resections, benign and malignant specimens according to tissue type in week 47 – week 51, during second lockdown.**

Legend to supplementary table 6: ^*^Ratio of the numbers observed in 2020 and the numbers observed in 2019 with, in brackets, the CI of this ratio, calculated using the standard error of a ratio of two independent Poisson distributed count variables. Red and green boxes show situations where the confidence interval of the ratio of measured and expected does not contain 100% and is considered statistically significantly different. Red and green numbers show a ratio of less than 85% or more than 115%, which might be clinically significant. na = not available (less than 20 specimens per week).

**Supplementary table 7. Summary of table 5 and supplementary tables 3-6. Periods with statistical significant decrease in numbers in 2020 as compared with 2019 specified for tissue type, procedure and dignity of diagnosis**

Legend to supplementary table 7: Red boxes show situations where all periods show a statistical significant decrease of numbers. Dark brown boxes show situations where 3-4 periods are affected. Light brown boxes show situations 2 periods are affected. Pink boxes show situations where one period is affected. Green boxes (n) show situations where none of the periods seem to be affected. Na= no data available. P = period.
